# Human tear fluid proteome dataset for usage as a spectral library and for protein modeling

**DOI:** 10.1016/j.dib.2019.103742

**Published:** 2019-03-07

**Authors:** Annika Guntermann, Simone Steinbach, Bettina Serschnitzki, Pia Grotegut, Sabrina Reinehr, Stephanie C. Joachim, Marc Schargus, Katrin Marcus, Caroline May

**Affiliations:** a Medizinisches Proteom-Center, Ruhr-University Bochum, Bochum, Germany; b Experimental Eye Research, University Eye Hospital, Ruhr-University Bochum, Bochum, Germany; c Department of Ophthalmology, University Hospital Duesseldorf, Heinrich-Heine-University Duesseldorf, Germany; d Asklepios Eye Hospital Nord-Heidberg, Hamburg, Germany

## Abstract

This article provides a detailed dataset of human tear fluid proteins. Samples were fractionated by sodium dodecyl sulfate (SDS) gel electrophoresis resulting in 48 fractions that were spiked with an indexed retention time (iRT) peptide standard. These data are based on a data-dependent acquisition (DDA) mass spectrometric approach and can be used for example as a spectral library for tear fluid proteome analysis by data-independent acquisition (DIA). Moreover, the provided data set can be used with optimized HPLC and mass spectrometric settings for proteins/peptides of interest. Besides these aspects, this dataset can serve as a protein overview for gene ontology enrichment analysis and for modeling and benchmarking of multiple signaling pathways associated with the ocular surface in healthy or disease stages. The mass spectrometry proteomics data from the described workflow have been deposited to the ProteomeXchange Consortium via the PRIDE partner repository with the dataset identifier PXD011075.

**Table:** Specifications table

Subject area	*Proteomics*
More specific subject area	*DIA based mass spectrometric analysis of human tear fluid*
Type of data	*Raw files, msf.files*
How data was acquired	*Mass spectrometry (Q Exactive HF mass spectrometer operated in data-dependent acquisition (DDA) mode performing HCD fragmentation)*
Data format	*Raw files, unfiltered*
Experimental factors	*Data were obtained by mass spectrometric DDA measurements of human tear fluid spiked with iRT peptides for use as a spectral library.*
Experimental features	*Tear fluid was fractionated via gel electrophoresis and in-gel digested resulting in 48 bands.*
Data source location	*Bochum, Germany (51°26*′*43.4*″*N 7°15*′*27.9*″*E)*
Data accessibility	*The data files are hosted in the public repository ProteomeXchange with identifier PXD011075.*
Related research article	*Barkovits K., Linden A., Galozzi S., Schilde L., Pacharra S., Mollenhauer B., Stoepel N., Steinbach S., May C., Uszkoreit J., Eisenacher M., Marcus K.: Characterization of Cerebrospinal Fluid via Data-Independent Acquisition Mass Spectrometry*[1] *Published in J Proteome Res. 2018*

**Table:** 

**Value of the data** The dataset supplies standard proteomic data based on human tear fluid measured in DDA mode. The samples were spiked with iRT peptides. • This spiked data set can be used as a spectral library for human tear fluid analysis in DIA mode for example in the context of ocular surface-related diseases. • Data can serve as an overview with detailed information for further gene ontology enrichment analysis. • It may help to find optimized parameters for identification of proteins/peptides of interest. • It can be applied for modeling and benchmarking of multiple signaling pathways associated with the eye.

## 1 Data

**Fig. 1:**
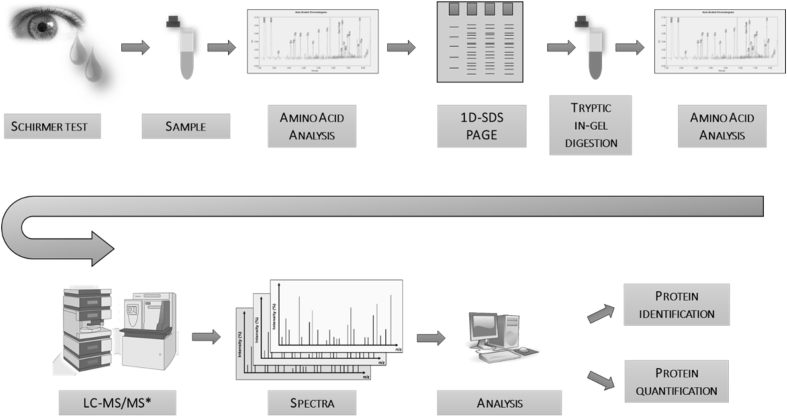
**Mass spectrometric analysis of human tear fluid.** This illustration shows a workflow overview of proteomic tear fluid examination. After tear fluid collection with a Schirmer Test strip and elution, protein concentration was determined by amino acid analysis. After that, an SDS gel (4–12% Bis-Tris) electrophoresis and staining followed. Then, protein lanes were fractioned, digested with trypsin and peptides extracted. Samples were again measured via amino acid analysis. In total, 48 fractions (12 per lane) were generated for nanoHPLC-ESI-MS/MS. (*modified image taken from http://planetorbitrap.com/, Thermo Fisher Scientific Inc., USA).

The here presented proteomic dataset offers mass spectrometry data files generated from human tear fluid (see Fig. 1). The tear fluid was collected with Schirmer test strips. Afterwards, proteins were eluted, the protein concentration determined and proteins separated by SDS gel electrophoresis. The resulting lanes were cut into single bands followed by an in-gel digestion with trypsin. After peptide extraction iRT peptides were added to each sample and the samples were measured with a data-dependent based mass spectrometric approach.

### 1.1 Experimental design, materials and methods

For a detailed workflow overview see Fig. 1.

#### 1.1.1 Sample collection

Tear fluid was collected via Schirmer test (Haag-Streit UK Ltd, United Kingdom) without anesthesia from 20 healthy individuals. During this process, a small filter strip was placed inside the lower lid of the right and left eye, respectively. Then individuals closed their eyes and tears were collected for 5 min. After that, strips were removed. Samples were pooled and tear fluid was eluted from the strips with 10 mL solution buffer containing phosphate buffered saline (Thermo Fisher Scientific Inc., USA), 0.1% (v/v) Triton-X-100 (AppliChem GmbH, Germany), and an EDTA-free protease inhibitor tablet (Roche Diagnostics GmbH, Germany). The incubation was performed at 4 °C continuous shaking overnight. After this, the supernatant was transferred, aliquoted into 1.5 mL reaction tubes and frozen at −80 °C until further usage. The remaining filter strips without solution were discarded.

#### 1.1.2 Amino acid analysis

Protein concentration of the tear fluid was determined via amino acid analysis. First, glass vials to be used for this technique were incubated in a muffle furnace (muffle furnace, Carbolite CWF 1100, USA) at 400 °C for 4 h to avoid contaminations. Each sample was analyzed in duplicate. In each clean glass vial, 4 μL of the eluted tear fluid was transferred, dried in a vacuum concentrator (RVC2-25CD plus) and placed in an evacuation vessel. After that, 400 μL 6 M hydrochloric acid and one phenol crystal were added. The evacuation was performed four times in alternation and samples were aerated with argon. The acidic gas phase hydrolysis was done at 150 °C for 1 h to cleave peptides into single amino acids. Then, peptide samples were evacuated again to remove the residual hydrochloric acid. Derivatization was proceeded by adding 30 μL of AccQ-Fluor borate buffer with the internal standard Norleucine and 10 μL AccQ-Fluor reagent (10 mM 6-aminoquinolyl-*N*-hydroxysuccinimidylcarbamate in acetonitrile), followed by an incubation at 55 °C for 10 min. Thereby, primary and secondary amines were converted to stable derivatives. These derivatives were separated on a C18 reversed-phase separation column (2.1 mm × 100 mm in length, Waters GmbH, Germany). Next, the hydrolyzed peptides were dissolved in 10 μL of 20 mM hydrochloric acid. For separation, a gradient system consisting of two solvents was used (solvent A: AccQ-Tag Ultra Eluent A, solvent B: AccQ-Tag Ultra Eluent B). To elute the derivatives, flow rate was set to 0.7 mL per minute with a column temperature of 55C and an increase of solvent A in the gradient (see Table 1). The amino acid derivatives were detected at an emission wavelength of 260 nm utilizing UV-spectrophotometry (Waters GmbH, Germany). Quantitative analysis was performed by applying different concentrations of an internal amino acid standard. In consideration of the volume and the molar mass of each amino acid, protein concentration of the tear fluid sample was calculated.

**Table 1:** Solvent gradient profile for the elution of amino acids.

Time [min]	% Eluent A	% Eluent B
0	99.9	0.1
0.54	99.9	0.1
5.74	90.9	0.1
7.74	78.8	21.2
8.04	40.4	59.6
8.05	10.0	90
8.64	10.0	90
8.73	99.9	0.1
9.50	99.9	0.1

#### 1.1.3 Sample preparation

400 mL of the pooled human tear fluid (concentration: 0.6 μg/μL) was lyophilized overnight (Alpha 2–4 LDplus, Martin Christ Gefriertrocknungsanlagen GmbH, Germany) and dissolved in 130 μL distilled water. 20 μL Bolt™ sample reducing agent (Thermo Fisher Scientific Inc., USA) as well as 50 μL 4× LDS sample buffer (pH 8.5) comprising 26.5 mM Tris HCl, 35.25 mM Tris Base, 2% LDS, 10% glycerol, 0.055 mM Coomassie blue G250, and 0.045 mM phenol red was added. The sample was mixed and centrifuged briefly. To achieve denaturation, samples were incubated for 10 min at 95 °C and 350 rpm in a thermomixer (Thermomixer comfort, Eppendorf AG, Germany).

#### 1.1.4 SDS gel electrophoresis and gel staining

60 μg protein per lane (4 lanes in total) was loaded onto a NuPAGE™ 4–12% Bis-Tris gel (Thermo Fisher Scientific Inc., USA). MES running buffer (pH 7.3) was composed of 50 mM MES, 50 mM Tris Base, 0.1% (v/v) SDS and 1 mM EDTA. Gel electrophoresis was performed according to the manufacturer's protocol (200 mA/200 W; voltage profile: 50 V for 15 min followed by 180 V for 50 min). The gel was then stained with Coomassie Brilliant Blue (SimpleBlue™ SafeStain, Thermo Fisher Scientific Inc., USA) in accordance with the manufacturer's instructions. Afterwards, the protein lanes were cut into single bands (in total 12 per lane) and each of them was transferred into a glass vial. Destaining and pH adjustment were achieved by incubating the gel pieces in 50 mM ammonium bicarbonate (Sigma-Aldrich Chemie GmbH, Germany). The washing solution was discarded after 10 min and another solution comprising of 50% (v/v) 50 mM ammonium bicarbonate and 50% (v/v) 100% acetonitrile (Merck KGaA, Germany) was added again for 10 min. Wash cycles were performed in repetition alternately four times. Gel pieces were then dried in a vacuum concentrator (Concentrator plus, Eppendorf AG, Germany).

#### 1.1.5 In-gel digestion and peptide extraction

Digestion was initiated by adding 6 μL of trypsin solution (0.033 μg/μL, Promega Corp., USA) to each fraction. During digestion, the samples were kept at 37 °C and 350 rpm overnight. The digestion was stopped and peptides were extracted by incubating the gel pieces two times for 15 min with 40 μL of a 1:1 solution containing 100% (v/v) acetonitrile and 0.1% (v/v) trifluoroacetic acid (Merck KGaA, Germany) in an ice-cooled ultrasonic bath (FB 15052, Fisher Scientific, United Kingdom). Finally, samples were transferred into glass vials, dried in the vacuum concentrator and resolved in 15 μL 0.1% (v/v) trifluoroacetic acid. Before nanoHPLC-MS/MS-analysis, samples were spiked with one injection volume equivalent of iRT standard peptides as described by the manufacturer (Biognosys AG, Switzerland)*.*

#### 1.1.6 NanoHPLC settings

The extracted peptides were first concentrated and then separated with an UltiMate™ 3000 RSLCnano system (Thermo Fisher Scientific Inc., USA) equipped with a capillary precolumn (100 μm × 2 cm, particle size 5 μm, pore size 100 Å; Thermo Fisher Scientific Inc., USA). After 7 min of desalting with 0.1% trifluoroacetic acid, peptides were eluted from the pre-column and on-line transferred to a reversed-phase analytical column (PepMap C18 75 μm × 50 cm, particle size 2 μm, pore size 100 Å; Thermo Scientific Inc., USA). Then, peptide separation was achieved by applying a stepwise 150 min gradient of solvent A (0.1% formic acid) and solvent B (84% acetonitrile, 0.1% formic acid) at 400 nL per minute flow rate (see Table 2). The temperature of the column oven was set at 60 °C. The concentration of solvent B increased from 5% to 60% within 127 min, followed by a washing step with 95% solvent B equilibrating for 5 min.

**Table 2:** Solvent gradient profile for elution of peptides.

Time [min]	% Eluent A	% Eluent B
0.00	95.00	5.00
7.00	95.00	5.00
15.00	85.00	15.00
122.00	60.00	40.00
127.00	40.00	60.00
128.00	5.00	95.00
133.00	5.00	95.00
138.00	95.00	5.00
150.00	95.00	5.00

#### 1.1.7 Mass spectrometry settings

The nanoHPLC system was directly connected to a nano-electrospray ionization source (Thermo Fisher Scientific Inc., USA) with 250 °C capillary temperature and a 1600 V spray voltage. Thereby, peptides were ionized by electrospray ionization and injected into a Q Exactive™ HF tandem mass spectrometer (Thermo Fisher Scientific Inc., USA). The instrument was operated in a data-dependent acquisition mode with the XCalibur software (Thermo Fisher Scientific Inc., USA). Mass spectra were acquired with a scan range from 350 to 1400 *m*/*z* and a full scan mode resolution set to 60,000 (AGC 3e6, 80 ms maximum injection time, 1.6 *m*/*z* wide isolation window). Fragment ions generated from the top 10 most intensive precursor ions were selected for higher-energy collisional dissociation (HCD) fragmentation at 28% NCE. Resulting fragment ions were examined in an Orbitrap mass analyzer acquiring resolving power of 30,000 at 100 *m*/*z* (AGC 1e6, 120 ms maximum injection time). Dynamic exclusion was utilized within 30 s to avoid a repeated selection of the same peptide. For internal recalibration, the lock mass polydimethylcyclosiloxane (445.120 *m*/*z*) was employed.
